# First-in-Human Drug-Eluting Balloon Treatment of Vulnerable Lipid-Rich Plaques: Rationale and Design of the DEBuT-LRP Study

**DOI:** 10.3390/jcm12185807

**Published:** 2023-09-06

**Authors:** Anna van Veelen, I. Tarik Küçük, Federico H. Fuentes, Yirga Kahsay, Hector M. Garcia-Garcia, Ronak Delewi, Marcel A. M. Beijk, Alexander W. den Hartog, Maik J. Grundeken, M. Marije Vis, José P. S. Henriques, Bimmer E. P. M. Claessen

**Affiliations:** 1 Heart Center, Department of Cardiology, Amsterdam UMC, University of Amsterdam, Amsterdam Cardiovascular Sciences, 1105 AZ Amsterdam, The Netherlands; a.vanveelen@amsterdamumc.nl (A.v.V.); i.t.kucuk@amsterdamumc.nl (I.T.K.); r.delewi@amsterdamumc.nl (R.D.); m.a.beijk@amsterdamumc.nl (M.A.M.B.); a.w.denhartog@amsterdamumc.nl (A.W.d.H.); m.j.grundeken@amsterdamumc.nl (M.J.G.); m.m.vis@amsterdamumc.nl (M.M.V.); j.p.henriques@amsterdamumc.nl (J.P.S.H.); 2 MedStar Washington Hospital Center, Washington, DC 20010, USA; federico.h.fuentes@medstar.net (F.H.F.); yirga.kahsay@medstar.net (Y.K.); hector.m.garciagarcia@medstar.net (H.M.G.-G.)

**Keywords:** vulnerable plaque, drug-coated balloon, intracoronary imaging, intravascular ultrasound, near-infrared spectroscopy, non-ST-segment elevation acute coronary syndrome

## Abstract

Patients with non-obstructive lipid-rich plaques (LRPs) on combined intravascular ultrasound (IVUS) and near-infrared spectroscopy (NIRS) are at high risk for future events. Local pre-emptive percutaneous treatment of LRPs with a paclitaxel-eluting drug-coated balloon (PE-DCB) may be a novel therapeutic strategy to prevent future adverse coronary events without leaving behind permanent coronary implants. In this pilot study, we aim to investigate the safety and feasibility of pre-emptive treatment with a PE-DCB of non-culprit non-obstructive LRPs by evaluating the change in maximum lipid core burden in a 4 mm segment (maxLCBImm4) after 9 months of follow up. Therefore, patients with non-ST-segment elevation acute coronary syndrome underwent 3-vessel IVUS-NIRS after treatment of the culprit lesion to identify additional non-obstructive non-culprit LRPs, which were subsequently treated with PE-DCB sized 1:1 to the lumen. We enrolled 45 patients of whom 20 patients (44%) with a non-culprit LRP were treated with PE-DCB. After 9 months, repeat coronary angiography with IVUS-NIRS will be performed. The primary endpoint at 9 months is the change in maxLCBImm4 in PE-DCB-treated LRPs. Secondary endpoints include clinical adverse events and IVUS-derived parameters such as plaque burden and luminal area. Clinical follow-up will continue until 1 year after enrollment. In conclusion, this first-in-human study will investigate the safety and feasibility of targeted pre-emptive PE-DCB treatment of LRPs to promote stabilization of vulnerable coronary plaque at risk for developing future adverse events.

## 1. Introduction

Ischemic heart disease is a major cause of morbidity and mortality [1]. Despite guideline-directed medical therapy for secondary prevention, patients remain at increased residual risk for repeat coronary events after percutaneous coronary intervention (PCI) for acute coronary syndrome (ACS). This is often caused by lesions other than the previously stented segment [2]. ACS is mostly precipitated by the rupture of a cholesterol-rich lipid core atheroma [3]. These lipid-rich plaques (LRPs) comprise specific high-risk features, such as a large lipid core, a thin fibrous cap and neovascularization, that increase the vulnerability to rupture and subsequently increase the risk of major adverse cardiovascular events (MACE) [4,5,6]. Half of patients undergoing PCI have residual vulnerable plaques [3]. The detection of these vulnerable LRPs may be of great importance for early prevention of ACS.

It is nearly impossible to detect LRPs using only coronary angiography (CAG) since most LRPs are angiographically mild with a diameter stenosis of less than 50% [7]. The addition of intracoronary imaging techniques allows for more profound plaque analysis. Near-infrared spectroscopy (NIRS) is a novel imaging technique that visualizes lipid components in the coronary artery wall. NIRS combined with intravascular ultrasound (IVUS-NIRS) enables additional analysis of plaque composition and structure. The lipid burden can be quantified as the lipid-core burden index (LCBI), which is a validated tool to detect plaques that are at high risk of rupture and causing subsequent MACE [4,8]. Whether MACE can be prevented by prophylactic treatment of LRPs, has not yet been established.

Local treatment with PCI using a bioresorbable vascular scaffold was investigated in the PROSPECT Absorb trial and has appeared to be safe, improve the minimal luminal area (MLA), reduce the lipid burden and potentially decrease the residual risk for MACE [9]. To overcome risks associated with stent or scaffold implantation, balloons coated with an anti-proliferative drug (drug-coated balloon, DCB) have been developed [10,11,12]. DCBs provide a selective pharmacological treatment that could halt the local atherosclerotic process and a mechanical treatment by low-pressure balloon inflation. A recent animal study showed that paclitaxel-eluting DCB (PE-DCB) treatment of non-obstructive plaques led to a reduction of inflammation and plaque burden [13]. In addition, DCBs are able to decrease the percentage of lipids of coronary plaque and improve fibrous cap thickness [14,15]. This suggests that PE-DCB may play an important role in the stabilization of coronary plaques and the prevention of coronary events. Therefore, we designed the First-in-Human Drug-Eluting Balloon Treatment of Vulnerable Lipid-Rich Plaques (DEBuT-LRP) pilot study to evaluate the safety and feasibility of PE-DCB as a prophylactic treatment for non-obstructive non-culprit LRPs in patients with non-ST-segment elevation ACS (NSTE-ACS). We aimed to determine the change in plaque characteristics after PE-DCB treatment of non-culprit LRPs, as measured with IVUS-NIRS. Our hypothesis is that PE-DCB will lead to a reduction of the LCBI after 9 months of follow-up without overt safety concerns. In this paper, we will present the rationale and design of this study.

## 2. Materials and Methods

### 2.1. Overview

**Figure 1:**
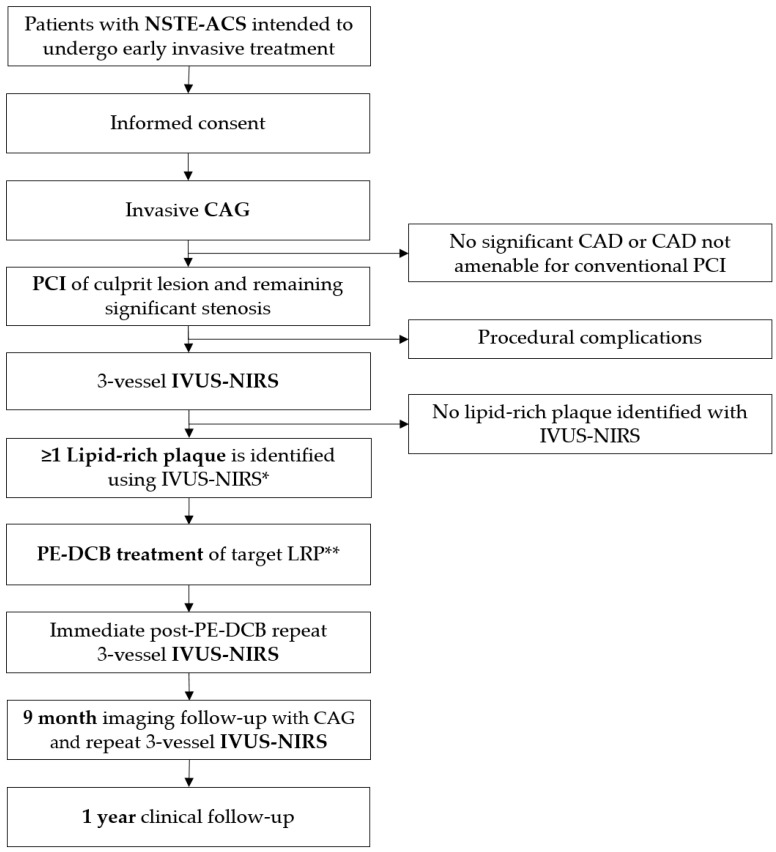
Flowchart of study protocol. ACS denotes acute coronary syndrome, CAD coronary artery disease, CAG coronary angiography, IVUS intravascular ultrasound, LRP lipid-rich plaque, NIRS near-infrared spectroscopy, PCI percutaneous coronary intervention, PE-DCB paclitaxel-eluting drug-coated balloon. * Defined as a maximum lipid-core burden index in a 4 mm segment (maxLCBImm4) of >325; ** in case multiple LRPs are detected, only one will be treated.

The DEBuT-LRP study (ClinicalTrials.gov identifier: NCT04765956) is a first-in-human, proof-of-concept, investigator-initiated, single-center, prospective single-arm interventional trial in consecutive NSTE-ACS patients. The aim of the study is to determine the change in plaque characteristics of non-obstructive non-culprit lipid-rich plaques, as measured with IVUS-NIRS, after treatment with a drug-eluting balloon in patients with NSTE-ACS. A flowchart of study procedures is provided in Figure 1. The Medical Ethics Committee on Human Research of the Amsterdam University Medical Centers, location AMC, approved the study protocol. The study is conducted in accordance with the declaration of Helsinki. This study was registered at ClinicalTrials.gov prior to starting under the identifier NCT04765956. Funding consisted of a private–public partnership grant provided by Health~Holland, with contributions from B. Braun Melsungen AG, Melsungen, Germany, and Infraredx, Inc., Bedford, MA, USA.

### 2.2. Patient Selection

Patients presenting with NSTE-ACS were screened for inclusion in the trial. All inclusion and exclusion criteria are provided in Table 1. Patients were eligible for inclusion when they had NSTE-ACS, according to the European Society of Cardiology guidelines [16,17], and an invasive imaging strategy (coronary angiogram) was chosen. Exclusion criteria included factors that hamper adequate image acquisition, such as the presence of a chronic total occlusion or previous coronary artery bypass grafting, or patient factors such as cardiogenic shock or ST-segment elevations that require immediate intervention. Informed consent was obtained before the coronary angiogram as mandated by the Medical Ethics Committee and in accordance with the declaration of Helsinki.

### 2.3. Imaging

When all inclusion criteria and none of the exclusion criteria were met, patients underwent CAG at baseline followed by PCI of the culprit lesion. After successful PCI of the culprit lesion in a native coronary artery, without significant peri-procedural complications, 3-vessel IVUS-NIRS was performed. Patients in whom no PCI was performed during the index procedure did not undergo 3-vessel IVUS NIRS and were withdrawn from the study. During IVUS-NIRS, arterial segments were scanned approximately 80–100 mm from the ostium. The amount of lipid-core plaque was quantified as the maximum LCBI within any 4 mm segment (maxLCBImm4). The presence of a maxLCBImm4 of >325 was the adopted definition for a ‘lipid-rich plaque’ from the large prospective PROSPECT II study, where the highest quartile of maxLCBImm4 of all non-culprit lesions in their cohort of almost 900 patients was used as the cut-off [6]. Initially, the often-used threshold of maxLCBImm4 >400 was used for the definition of LRP, as used in the LRP study [4]. However, this threshold is derived from smaller retrospective studies and was substituted by the PROSPECT II derived definition [18,19]. To differentiate between single or multiple LRPs within the same coronary artery a 5 mm lipid-free margin was taken into account. All images will be adjudicated in a dedicated core lab for the primary endpoint analysis. Directly after the PE-DCB treatment of the identified target LRP, a post-procedural IVUS-NIRS of the PE-DCB-treated vessel was performed for data acquisition only.

**Figure 2:**
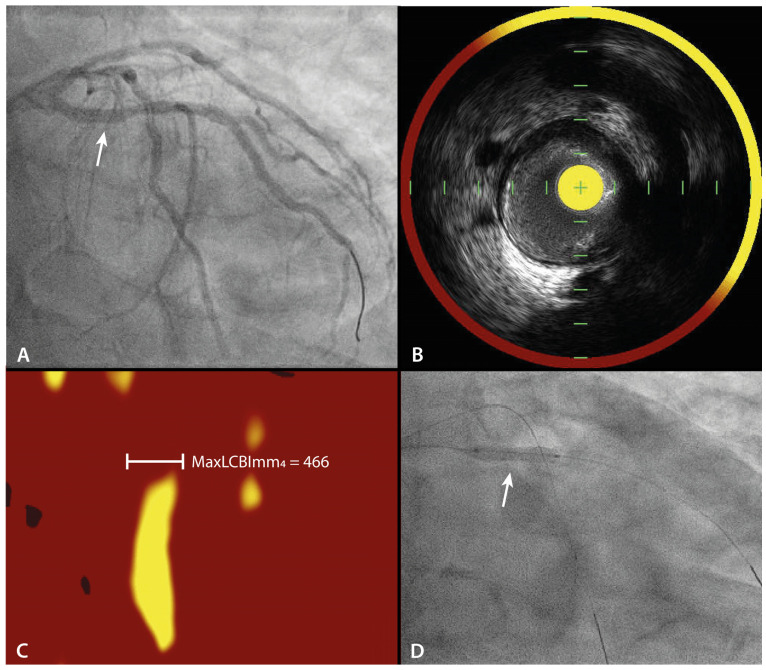
Case example. (**A**) Depicts the coronary angiogram, wherein the proximal circumflex artery, as highlighted with the white arrow, a lipid-rich plaque was observed with intravascular ultrasound (IVUS, (**B**)) and near-infrared spectroscopy (NIRS, (**C**)). (**D**) This lesion was subsequently treated with paclitaxel-eluting drug-coated balloon (PE-DCB) as highlighted with the white arrow.

At 9-month follow-up, a repeat CAG with 3-vessel IVUS-NIRS was performed to assess the primary endpoint. As exploratory analyses, IVUS-NIRS data of untreated LRPs will be used to assess the natural course of a LRP and to compare with the possible treatment effect of PE-DCB. Figure 2 demonstrates a case example of the acquired baseline imaging.

### 2.4. Investigational Treatment

If during 3-vessel IVUS-NIRS a non-culprit LRP was detected, this was subsequently treated with a PE-DCB. LRPs located in the left main artery were not treated. If more than 1 LRP was detected, only one lesion was determined to be treated as per the operators’ discretion with the instruction that this should preferably be the lesion with the highest maxLCBImm4. The other LRPs were not treated with PE-DCB.

After PCI, the target LRP was treated with the SeQuent^®^ Please NEO (B. Braun, Melsungen, Germany) PE-DCB catheter. The SeQuent^®^ Please Neo is polymer-free and consists of a standard percutaneous transluminal angioplasty balloon catheter, that is coated with the antimitotic drug paclitaxel at a dose of 3 microgram/mm^2^ and Iopromide as an excipient. The product is CE-marked (2015) and widely used. The PE-DCB catheter was previously proven to be clinically effective for in-stent restenosis, de novo coronary artery disease, small vessel disease and bifurcation lesions [10,12]. As we aimed to treat non-obstructive lesions, no specific lesion preparation prior to PE-DCB treatment was required in this particular subset of coronary lesions. The balloon catheter was sized 1:1 according to lumen diameter as derived from IVUS. The balloon was inflated at nominal pressure (6–8 ATM) during a period of minimally 60 s, but preferably 90–120 s. 

Bail-out drug-eluting stent placement of the PE-DCB-treated lesion was allowed in case of PE-DCB treatment failure, according to the following criteria: (1) thrombolysis in myocardial infarction (TIMI) flow of <3 that persists after administration of vasoactive drugs; (2) coronary dissection of ≥Type C, according to The National Heart, Lung and Blood Institute (NHLBI).

### 2.5. Imaging Analysis

The culprit lesion, the treated LRP and the PE-DCB inflation were angiographically recorded in two orthogonal views. All angiograms and IVUS-NIRS images will be analyzed by an independent Angiographic and NIRS-IVUS Core Laboratory (MedStar Cardiovascular Research Network, Angiographic and Invasive Imaging Core Lab, Washington, DC, USA). This core laboratory will analyze the angiograms using CAAS Workstation Software (version 8.5, Pie Medical Imaging, Maastricht, the Netherlands) and IVUS-NIRS using QIVUS Software (version 3.1.12.0, Medis Medical Imaging Systems, Leiden, the Netherlands) and QCU-CMS^®^ (v4.69, Leiden University Medical Center and MEDIS, Leiden, the Netherlands). A co-registration of the IVUS-NIRS scan and the angiogram will be made with the use of visible landmarks such as side branches or stents. The LCBI of the LRP will be calculated automatically by the software, as well as the maxLCBImm4, by dividing the yellow pixels by all valid pixels in the area of interest. The maxLCBImm4 of the treated LRP will be compared between baseline and 9 months follow-up and the absolute difference in units will be calculated, as well as the relative difference in percentage (%).

After semi-automated border detection of the coronary lumen at the IVUS images, the plaque burden of the LRP will be calculated, defined as (plaque area = external elastic membrane area − lumen area)/external elastic membrane area × 100%, as well as the plaque volume which is defined as the plaque area multiplied by the lesion length and minimum lumen area (MLA) in mm^2^.

### 2.6. Study Endpoints

The main study endpoint is the change in maxLCBImm4 as measured with IVUS-NIRS from baseline to 9 months follow-up in identified LRPs that are treated with PE-DCB. Secondary endpoints include the change in maxLCBImm4 after 9 months follow up in additional LRPs that are not treated with PE-DCB, safety endpoints including flow-limiting dissection necessitating bail-out stent implantation, peri-procedural myocardial infarction, LRP lesion failure, patient-oriented composite outcomes defined as all-cause mortality, myocardial infarction, or any repeat revascularization up to one-year follow-up, and additional IVUS-NIRS lesion characteristics (plaque burden and MLA). Study endpoints are summarized in Table 2.

Cardiovascular death is defined according to the ARC-2 criteria as all deaths related to a cardiovascular cause or procedure, or when the cause is undetermined [20]. LRP lesion failure is defined as cardiac death, myocardial infarction, or ischemia-driven revascularization related to an identified non-culprit LRP lesion up to one year of follow-up. Acute myocardial infarction is defined according to the Fourth Universal Definition of Myocardial Infarction [21]. Peri-procedural myocardial infarction has occurred when a >5- or >10-fold increase of pre-procedural Troponin T is observed, with at least one of the following: new ischemic ECG changes; development of new pathological Q waves; imaging evidence of loss of viable myocardium that is presumed to be new and in a pattern consistent with an ischemic etiology; angiographic findings consistent with a procedural flow-limiting complication such as coronary dissection, occlusion of a major epicardial artery or graft, side-branch occlusion–thrombus, disruption of collateral flow or distal embolization. MACE is defined as a composite of cardiac death, myocardial infarction, and ischemia-driven target lesion revascularization. And lastly, ischemia-driven target lesion revascularization is defined as any repeat revascularization of the target lesion (i.e., the corresponding coronary segment that was treated during index PCI), prompted by ischemic symptoms or objective evidence of ischemia.

### 2.7. Follow-Up

All enrolled patients will undergo follow-up by telephone at 1 month and 1 year. Patients who did not undergo PE-DCB treatment of a LRP after the index PCI will undergo follow-up by telephone at 9 months. Patients with a LRP and PE-DCB treatment will undergo clinical follow-up by an outpatient visit including repeat CAG after 9 months. During all follow-up moments, clinical evaluation of the angina class using the Canadian Cardiovascular Score (CCS) for angina pectoris [22], as well as the New York Heart Association (NYHA) class for heart failure [23] will be performed. All medications will be registered, with a specific focus on lipid-lowering medication.

### 2.8. Statistical Considerations

Continuous baseline demographic and clinical variables will be presented in terms of percentiles (e.g., median, 25th, and 75th percentile or mean with standard deviation), while categorical variables are summarized in terms of frequencies and percentages. All *p*-values will be two-sided and a *p*-value of <0.05 will be considered statistically significant. The primary endpoint will be core-lab adjudicated and analyzed on both the lesion-level and the patient-level using a paired samples test. Missing data will be excluded from the analysis. The secondary endpoints will be summarized in frequencies and percentages.

A total of 40 patients undergoing percutaneous coronary intervention for NSTE-ACS, including unstable angina and non-ST-segment elevation myocardial infarction will be consented. Based upon the previous PROSPECT and LRP trials [3,4], approximately 50% of patients are expected to have an average of 2 non-culprit LRPs. Therefore, an expected 20 patients with 40 non-culprit LRPs will be included in this single-arm study. Patients found to have no non-culprit LRPs were followed in a parallel registry. Given the lack of any comparable data on direct pharmacological treatment of LRPs, no formal power calculation can be made. If the observed rate of LRP is found to be lower than 50%, patient inclusion will continue until 20 patients with LRP have been treated with PE-DCB.

### 2.9. Safety

Adverse events are defined as any undesirable experience occurring to a participant during the study that is considered related to the diagnostic procedures (IVUS-NIRS) or the experimental intervention (PE-DCB treatment of LRP). We will record all adverse events that are reported spontaneously by the participant, or when observed by the investigator or staff. A serious adverse event is defined according to the Central Committee on Research Involving Human Subjects (CCMO) as any untoward medical experience or effect that results in death; is life threatening (at the time of the event); requires hospitalization or prolongation of existing inpatients’ hospitalization; results in persistent or significant disability or incapacity; and any other important medical event that did not result in any of the outcomes listed above due to medical or surgical intervention but could have been based upon appropriate judgment by the investigator. An elective hospital admission will not be considered a serious adverse event. Adverse events are reported to the Ethics Committee through an annual line listing. Serious adverse events related to the study procedures are reported within 7 days, whereas serious adverse events not related to the study procedures are reported to the Ethics Committee through an annual line listing.

## 3. Current Status

Between January 2021 and September 2022, 65 patients consented to participate in this study, of which twenty did not undergo PCI after the diagnostic CAG, either due to multi-vessel disease or due to the absence of obstructive coronary artery disease. These patients were therefore not assessed with IVUS-NIRS. A total of 45 patients underwent IVUS-NIRS after PCI of whom 20 patients (44%) underwent additional PE-DCB treatment of a LRP. A total of six patients did have an additional LRP but did not undergo PE-DCB treatment due to LRP location (i.e., LRP in left main or in-stent). A total of 19 patients had no LRP. Nine-month IVUS-NIRS follow-up was completed in May 2023 and 1-year clinical follow-up will be finished by September 2023. Baseline characteristics of the participants are displayed in Table 3.

## 4. Discussion

The DEBuT-LRP study will be the first-in-human clinical study to investigate the impact of PE-DCB treatment on the maxLCBImm4 of LRPs. This study will evaluate whether prophylactic treatment of LRPs by PE-DCB leads to plaque stabilization by reducing the lipid burden. After the completed PROSPECT Absorb trial [9], the prematurely halted PECTUS trial [24] and the ongoing PREVENT trial (ClinicalTrials.gov Identifier NCT02316886), this will be the fourth study investigating prophylactic local treatment of LRPs, and the first study to investigate local treatment with PE-DCB.

### 4.1. Prognostic Impact of a Lipid-Rich Plaque

The PROSPECT, PROSPECT II and LRP studies have investigated the incidence and prognostic impact of the presence of a LRP in large cohorts of patients undergoing PCI [3,4,6]. In PROSPECT, it was found that half of the patients had an average of two vulnerable plaques per patient (i.e., thin-cap fibro-atheromas according to the study definition) [3]. A total of 20.4% MACE occurred in the total cohort of 697 patients during a follow-up period of 3.4 years. The most important plaque-related predictors of MACE were a MLA of <4 mm^2^, a plaque burden of >70%, and thin-cap fibroatheromas. The LRP study analyzed IVUS-NIRS images of more than 1200 patients after PCI of a culprit lesion and performed clinical follow-up for at least 2 years [4]. The 2-year risk of non-culprit MACE was 9%. Patients with LRP, according to study definition plaques with maxLCBImm4 of >400, demonstrated a two-fold increased risk for MACE than patients without LRP. On a plaque level, if a segment demonstrated a maxLCBImm4 of >400, there was an adjusted risk of 3.4 to cause MACE. In PROSPECT II, a 4-year non-culprit MACE rate of 8% was observed in a cohort of 898 patients [6]. Lesions with high plaque burden (≥70%) and maxLCBImm4 >325 had a 4-year MACE rate of 7% and patients with one or more of those lesions had a 4-year MACE rate of 13%. Thus, we may conclude from these aforementioned studies, together comprising a total of almost 3000 patients, that the risk of non-culprit MACE after PCI is limited, but significantly increased when a LRP is present. 

### 4.2. Systemic Treatment vs. Local Treatment

The obvious treatment strategy to reduce the residual risk of recurrent events after PCI or ACS is a medical therapy that targets the reduction of risk factors. Several studies have investigated the effect of aggressive lipid lowering with statins and proprotein convertase subtilisin kexin type 9 (PCSK9 inhibitors) on plaque stabilization and, more extensively, on clinical outcomes [25,26]. Early initiation of PCSK9 inhibitors after ACS is probably beneficial to clinical outcomes [27]. Intensified lipid lowering with PCSK9 inhibitors on top of maximally tolerated statin therapy leads to significant plaque stabilization, with reduced plaque burden and increased fibrous cap thickness as assessed with IVUS or optical coherence tomography [28,29,30]. The PACMAN-AMI trial showed a greater decrease of maxLCBImm4 in the group treated with PCSK9 inhibitors as compared with the placebo group (−79.42 vs. −37.60) [30].

Moreover, the anti-inflammatory agent colchicine reduces the residual atherosclerotic risk as well in patients with chronic coronary artery disease [31]. No intracoronary imaging studies on the effect of colchicine on plaque regressions have been performed yet, but with coronary computed tomography angiography (CCTA) it was observed that colchicine on top of optimal medical therapy was able to reduce the plaque burden after 1 year, compared to optimal medical therapy alone [32]. Nonetheless, although this aforementioned medical therapy leads to plaque regression and subsequent reduction of clinical events, patients often have important systemic side-effects reducing adherence and still have a residual risk of around 5–10% of MACE according to the large trials [24,25,31]. Therefore, the addition of local therapy on top of these medical therapy options seems rectified to further reduce the risk for repeat coronary events after ACS. 

### 4.3. Drug-Eluting Stent vs. Drug-Eluting Balloon

As mentioned above, the DEBuT-LRP trial will be the fourth study investigating the local treatment of LRPs, after PROSPECT Absorb [9], PECTUS [23] and the ongoing PREVENT trial (ClinicalTrials.gov Identifier NCT02316886). In PROSPECT Absorb, patients from PROSPECT II who had vulnerable plaques, defined as plaque burden ≥65%, were randomized between PCI of the vulnerable plaque or optimal medical therapy alone [9]. PCI was performed with a bioresorbable vascular scaffold after pre-dilation with a non-compliant balloon, sized 1:1 to the reference vessel lumen according to IVUS. Subsequently, the scaffold was sized after intracoronary nitroglycerine and was post-dilated after deployment at high pressure (>16 atmospheres) aiming for <10% residual stenosis. The study found that PCI of LRPs was safe and led to an enlargement of the MLA, a reduction of the maxLCBImm4 as measured with NIRS and a reduction of the plaque burden as measured with IVUS at 25-month follow-up CAG.

In our study, we achieved local drug delivery by using the nominal pressure, precisely sized to the true lumen instead of the reference lumen. Additionally, since we use PE-DCB and no stent or scaffold, stent-related complications such as stent thrombosis or in-stent restenosis can be prevented.

The eluted drug paclitaxel is known for its anti-inflammatory properties. In a recent animal study, Chowdhury et al. tested the use of paclitaxel-coated balloons on experimental non-obstructive atherosclerotic plaques in the aorta of cholesterol-fed rabbits [13]. Compared with plain balloon angioplasty and sham balloon angioplasty, paclitaxel-coated balloons were able to reduce the plaque burden and inflammation and increase the MLA as assessed with IVUS and near-infrared fluorescence. In addition, clinical cohort studies in humans demonstrated that paclitaxel-coated balloons are able to decrease the percentage of fat and improve fibrous cap thickness [14,15]. Therefore, we believe that adequate local drug delivery by PE-DCB, without traumatic mechanic pressure, will lead to a reduction of the lipid core and stabilization of the plaque. 

## 5. Conclusions

In conclusion, the DEBuT-LRP trial will be the first-in-human clinical study to investigate the pre-emptive treatment of LRPs with PE-DCB in patients with NSTE-ACS, as guided by IVUS-NIRS. The study will contribute to the understanding of whether local treatment of LRPs, on top of guideline-directed medical therapy, will lead to better plaque stabilization, potentially leading to a reduced risk of future acute coronary events.

## Figures and Tables

**Table 1:** Inclusion and exclusion criteria.

Inclusion Criteria
(1) Presence of NSTE-ACS (including non-ST-segment elevation MI and UA);
(2) An invasive revascularization strategy with PCI is chosen.
**Exclusion Criteria**
*Angiographic exclusion criteria*
(1) Previous coronary artery bypass grafting;
(2) Presence of a chronic total occlusion;
(3) Too many (complex) coronary lesions requiring staged PCI procedure(s);
(4) Procedural complication of the index PCI.
*Clinical exclusion criteria*
(1) Unstable patients (cardiogenic shock, need for intubation, need for inotropes);
(2) ST-segment elevations on the ECG requiring immediate primary PCI;
(3) Body weight > 250 kg;
(4) Known renal insufficiency (eGFR < 30 mL/min/1.73 m^2^ or subject on dialysis);
(5) Hypersensitivity or allergy to contrast with inability to properly pre-hydrate;
(6) Presence of a comorbid condition with a life expectancy of less than one year;
(7) Participation in another trial;
(8) Subject belongs to a vulnerable population (per the investigator’s judgment, e.g., subordinate hospital staff);
(9) Subject is unable to read or write.

MI denotes myocardial infarction, NSTE-ACS non-ST-segment elevation acute coronary syndrome, PCI percutaneous coronary intervention and UA unstable angina.

**Table 2:** Study endpoints.

Primary Endpoint
Change in maxLCBImm4 between baseline and 9 months follow-upin PE-DCB treated LRPs.
**Secondary Endpoints**
Change in maxLCBImm4 between baseline and 9 months follow-up in not PE-DCB treated LRPs; Flow-limiting dissection necessitating bail-out stent implantation; Peri-procedural myocardial infarction; LRP lesion failure, defined as cardiac death, myocardial infarction, or ischemia-driven revascularization related to an identified non-culprit LRP lesion up to one-year follow-up; Patient-oriented composite outcomes, defined as all-cause mortality, myocardial infarction, or any repeat revascularization up to one-year follow-up; Additional IVUS + NIRS lesion characteristics (plaque volume, minimal lumen area).

IVUS denotes intravascular ultrasound, LRP lipid-rich plaque, maxLCBImm4 maximum lipid core burden index in a 4 mm segment, NIRS near-infrared spectroscopy and PE-DCB paclitaxel-eluting drug-coated balloon.

**Table 3:** Baseline characteristics.

	PE-DCB (*n* = 20)
Age, (years)	66 (55–73)
Male	18 (90%)
Body mass index, (kg/m^2^)	26.4 (23.3–36.4) *
Current tobacco use	3 (15%) **
Chronic obstructive pulmonary disease	1 (5%)
Diabetes mellitus	1 (5%)
Hypertension	11 (55%)
Hypercholesterolemia	12 (60%)
Family history of coronary artery disease	5 (39%) ***
Prior myocardial infarction	3 (15%)
Prior percutaneous coronary intervention	3 (15%)
Prior bypass graft surgery	0 (0%)
Prior stroke	1 (5%)
Laboratory measures	
Total cholesterol, (mmol/L)	5.0 (4.2–6.4) ****
High-density lipoprotein, (mmol/L)	1.1 (1.0–1.3) *****
Low-density lipoprotein, (mmol/L)	2.8 (2.2–4.0) *****
Triglycerides, (mmol/L)	1.9 (1.2–2.6) *****
Hemoglobin, (mmol/L)	9.3 (8.7–10.1)
Leucocytes, (×10^9^/L)	7.7 (6.2–11.4) ******
Thrombocytes, (×10^9^/L)	204 (188–221)
Lipid-lowering medication prior to admission	9 (45%)
Statin	9 (45%)
Ezetimibe	0 (0%)
PCSK9 inhibitor	0 (0%)

Numbers are presented as median (interquartile range) or numbers (percentages). PCSK9 denotes proprotein convertase subtilisin/kexin type 9. * Data available from 11 patients. ** Data available from 15 patients. *** Data available from 13 patients. **** Data available from 7 patients. ***** Data available from 6 patients. ****** Data available from 19 patients.

## Data Availability

The data can be made available upon reasonable request.

## References

[1] Sanchis-Gomar F., Perez-Quilis C., Leischik R., Lucia A. (2016). Epidemiology of coronary heart disease and acute coronary syndrome. Ann. Transl. Med..

[2] Cutlip D., Chhabra A., Baim D. (2004). Beyond restenosis: Five-year clinical outcomes from second-generation coronary stent trials. Circulation.

[3] Stone G.W., Maehara A., Lansky A.J., De Bruyne B., Cristea E., Mintz G.S., Mehran R., McPherson J., Farhat N., Marso S.P. (2011). A prospective natural-history study of coronary atherosclerosis. N. Engl. J. Med..

[4] Waksman R., Di Mario C., Torguson R., Ali Z.A., Singh V., Skinner W.H., Artis A.K., Cate T.T., Powers E., Kim C. (2019). Identification of patients and plaques vulnerable to future coronary events with near-infrared spectroscopy intravascular ultrasound imaging: A prospective, cohort study. Lancet.

[5] van Veelen A., van der Sangen N.M.R., Delewi R., Beijk M.A.M., Henriques J.P.S., Claessen B.E.P.M. (2022). Detection of Vulnerable Coronary Plaques Using Invasive and Non-Invasive Imaging Modalities. J. Clin. Med..

[6] Erlinge D., Maehara A., Ben-Yehuda O., Bøtker H.E., Maeng M., Kjøller-Hansen L., Engstrøm T., Matsumura M., Crowley A., Dressler O. (2021). Identification of vulnerable plaques and patients by intracoronary near-infrared spectroscopy and ultrasound (PROSPECT II): A prospective natural history study. Lancet.

[7] Kolodgie F.D., Virmani R., Burke A.P., Farb A., Weber D.K., Kutys R., Finn A.V., Gold H.K. (2004). Pathologic assessment of the vulnerable human coronary plaque. Heart (Br. Card. Soc.).

[8] Gardner C.M., Tan H., Hull E.L., Lisauskas J.B., Sum S.T., Meese T.M., Jiang C., Madden S.P., Caplan J.D., Burke A.P. (2008). Detection of lipid core coronary plaques in autopsy specimens with a novel catheter-based near-infrared spectroscopy system. JACC Cardiovasc. Imaging.

[9] Stone G.W., Maehara A., Ali Z.A., Held C., Matsumura M., Kjøller-Hansen L., Bøtker H.E., Maeng M., Engstrøm T., Wiseth R. (2020). Percutaneous Coronary Intervention for Vulnerable Coronary Atherosclerotic Plaque. J. Am. Coll. Cardiol..

[10] Baan J., Claessen B.E., Dijk K.B.V., Vendrik J., van der Schaaf R.J., Meuwissen M., van Royen N., Marcel Gosselink A.T., van Wely M.H., Dirkali A. (2018). A Randomized Comparison of Paclitaxel-Eluting Balloon Versus Everolimus-Eluting Stent for the Treatment of Any In-Stent Restenosis: The DARE Trial. JACC Cardiovasc. Interv..

[11] Jeger R.V., Farah A., Ohlow M.-A., Mangner N., Möbius-Winkler S., Leibundgut G., Weilenmann D., Wöhrle J., Richter S., Schreiber M. (2018). Drug-coated balloons for small coronary artery disease (BASKET-SMALL 2): An open-label randomised non-inferiority trial. Lancet.

[12] Liu L., Liu B., Ren J., Hui G., Qi C., Wang J. (2018). Comparison of drug-eluting balloon versus drug-eluting stent for treatment of coronary artery disease: A meta-analysis of randomized controlled trials. BMC Cardiovasc. Disord.

[13] Chowdhury M.M., Singh K., Albaghdadi M.S., Khraishah H., Mauskapf A., Kessinger C.W., Osborn E.A., Kellnberger S., Piao Z., Cardenas C.L.L. (2020). Paclitaxel Drug-Coated Balloon Angioplasty Suppresses Progression and Inflammation of Experimental Atherosclerosis in Rabbits. JACC Basic Transl. Sci..

[14] Ann S.H., Balbir Singh G., Lim K.H., Koo B.K., Shin E.S. (2016). Anatomical and Physiological Changes after Paclitaxel-Coated Balloon for Atherosclerotic De Novo Coronary Lesions: Serial IVUS-VH and FFR Study. PLoS ONE.

[15] Her A.-Y., Shin E.-S., Chung J.-H., Kim Y.H., Garg S., Lee J.M., Doh J.-H., Nam C.-W., Koo B.-K. (2019). Plaque modification and stabilization after paclitaxel-coated balloon treatment for de novo coronary lesions. Heart Vessel..

[16] Collet J.-P., Thiele H., Barbato E., Barthélémy O., Bauersachs J., Bhatt D.L., Dendale P., Dorobantu M., Edvardsen T., Folliguet T. (2021). 2020 ESC Guidelines for the management of acute coronary syndromes in patients presenting without persistent ST-segment elevation. Eur. Heart J..

[17] Byrne R.A., Rossello X., Coughlan J.J., Barbato E., Berry C., Chieffo A., Claeys M.J., Dan G.-A., Dweck M.R., Galbraith M. (2023). 2023 ESC Guidelines for the management of acute coronary syndromes: Developed by the task force on the management of acute coronary syndromes of the European Society of Cardiology (ESC). Eur. Heart J..

[18] Madder R.D., Goldstein J.A., Madden S.P., Puri R., Wolski K., Hendricks M., Sum S.T., Kini A., Sharma S., Rizik D. (2013). Detection by near-infrared spectroscopy of large lipid core plaques at culprit sites in patients with acute ST-segment elevation myocardial infarction. JACC Cardiovasc. Interv..

[19] Madder R.D., Husaini M., Davis A.T., Van Oosterhout S., Harnek J., Götberg M., Erlinge D. (2015). Detection by near-infrared spectroscopy of large lipid cores at culprit sites in patients with non-ST-segment elevation myocardial infarction and unstable angina. Catheter. Cardiovasc. Interv. Off. J. Soc. Card. Angiogr. Interv..

[20] Garcia-Garcia H.M., McFadden E.P., Farb A., Mehran R., Stone G.W., Spertus J., Onuma Y., Morel M.-A., van Es G.-A., Zuckerman B. (2018). Standardized End Point Definitions for Coronary Intervention Trials: The Academic Research Consortium-2 Consensus Document. Circulation.

[21] Thygesen K., Alpert J.S., Jaffe A.S., Chaitman B.R., Bax J.J., Morrow D.A., White H.A. (2018). Fourth Universal Definition of Myocardial Infarction (2018). Circulation.

[22] Campeau L. (1976). Letter: Grading of angina pectoris. Circulation.

[23] The Criteria Committee of the New York Heart Association (1994). Nomenclature and Criteria for Diagnosis of Diseases of the Heart and Great Vessels.

[24] Mol J.Q., Bom M.J., Damman P., Knaapen P., van Royen N. (2020). Pre-Emptive OCT-Guided Angioplasty of Vulnerable Intermediate Coronary Lesions: Results from the Prematurely Halted PECTUS-Trial. J. Interv. Cardiol..

[25] Sabatine M.S., Giugliano R.P., Keech A.C., Honarpour N., Wiviott S.D., Murphy S.A., Kuder J.F., Wang H., Liu T., Wasserman S.M. (2017). Evolocumab and Clinical Outcomes in Patients with Cardiovascular Disease. N. Engl. J. Med..

[26] Schwartz G.G., Steg P.G., Szarek M., Bhatt D.L., Bittner V.A., Diaz R., Edelberg J.M., Goodman S.G., Hanotin C., Harrington R.A. (2018). Alirocumab and Cardiovascular Outcomes after Acute Coronary Syndrome. N. Engl. J. Med..

[27] Ferri N., Ruscica M., Lupo M.G., Vicenzi M., Sirtori C.R., Corsini A. (2022). Pharmacological rationale for the very early treatment of acute coronary syndrome with monoclonal antibodies anti-PCSK9. Pharmacol. Res..

[28] Nicholls S.J., Kataoka Y., Nissen S.E., Prati F., Windecker S., Puri R., Hucko T., Aradi D., Herrman J.-P.R., Hermanides R.S. (2022). Effect of Evolocumab on Coronary Plaque Phenotype and Burden in Statin-Treated Patients Following Myocardial Infarction. JACC Cardiovasc. Imaging.

[29] Nicholls S.J., Puri R., Anderson T., Ballantyne C.M., Cho L., Kastelein J.J., Koenig W., Somaratne R., Kassahun H., Yang J. (2016). Effect of Evolocumab on Progression of Coronary Disease in Statin-Treated Patients: The GLAGOV Randomized Clinical Trial. Jama.

[30] Räber L., Ueki Y., Otsuka T., Losdat S., Häner J.D., Lonborg J., Fahrni G., Iglesias J.F., van Geuns R.-J., Ondracek A.S. (2022). Effect of Alirocumab Added to High-Intensity Statin Therapy on Coronary Atherosclerosis in Patients With Acute Myocardial Infarction: The PACMAN-AMI Randomized Clinical Trial. Jama.

[31] Nidorf S.M., Fiolet A.T., Mosterd A., Eikelboom J.W., Schut A., Opstal T.S., The S.H.K., Xu X.-F., Ireland M.A., Lenderink T. (2020). Colchicine in Patients with Chronic Coronary Disease. N. Engl. J. Med..

[32] Vaidya K., Arnott C., Martínez G.J., Ng B., McCormack S., Sullivan D.R., Celermajer D.S., Patel S. (2018). Colchicine Therapy and Plaque Stabilization in Patients With Acute Coronary Syndrome: A CT Coronary Angiography Study. JACC Cardiovasc. Imaging.

