# Correction: van Veelen et al. First-in-Human Drug-Eluting Balloon Treatment of Vulnerable Lipid-Rich Plaques: Rationale and Design of the DEBuT-LRP Study. *J. Clin. Med.* 2023, *12*, 5807

**DOI:** 10.3390/jcm13051479

**Published:** 2024-03-04

**Authors:** Anna van Veelen, I. Tarik Küçük, Federico H. Fuentes, Yirga Kahsay, Hector M. Garcia-Garcia, Ronak Delewi, Marcel A. M. Beijk, Alexander W. den Hartog, Maik J. Grundeken, M. Marije Vis, José P. S. Henriques, Bimmer E. P. M. Claessen

**Affiliations:** 1 Heart Center, Department of Cardiology, Amsterdam UMC, University of Amsterdam, Amsterdam Cardiovascular Sciences, 1105 AZ Amsterdam, The Netherlandsi.t.kucuk@amsterdamumc.nl (I.T.K.); r.delewi@amsterdamumc.nl (R.D.); m.a.beijk@amsterdamumc.nl (M.A.M.B.); a.w.denhartog@amsterdamumc.nl (A.W.d.H.); m.j.grundeken@amsterdamumc.nl (M.J.G.); m.m.vis@amsterdamumc.nl (M.M.V.); j.p.henriques@amsterdamumc.nl (J.P.S.H.); 2 MedStar Washington Hospital Center, Washington, DC 20010, USA; federico.h.fuentes@medstar.net (F.H.F.); yirga.kahsay@medstar.net (Y.K.); hector.m.garciagarcia@medstar.net (H.M.G.-G.)

## 1. Text Correction

In the original publication [1], there was a mistake in Section 2.8. Statistical Considerations, Paragraph 1. The corrected version of Section 2.8. Statistical Considerations, Paragraph 1 appears below:

Continuous baseline demographic and clinical variables will be presented in terms of percentiles (e.g., median, 25th, and 75th percentile or mean with standard deviation), while categorical variables are summarized in terms of frequencies and percentages. All *p*-values will be two-sided and a *p*-value of <0.05 will be considered statistically significant. The primary endpoint will be core-lab adjudicated and analyzed on both the lesion-level and the patient-level using a paired samples test. Missing data will be excluded from the analysis. The secondary endpoints will be summarized in frequencies and percentages.

## 2. Error in Table

In the original publication [1], there was a mistake in Table 3, as published. The corrected version of Table 3 appears below.

The authors state that the scientific conclusions are unaffected. This correction was approved by the Academic Editor. The original publication has also been updated.

## Figures and Tables

**Table 3:** Baseline characteristics.

	PE-DCB (*n* = 20)
Age, (years)	66 (55–73)
Male	18 (90%)
Body mass index, (kg/m^2^)	26.4 (23.3–36.4) *
Current tobacco use	3 (15%) **
Chronic obstructive pulmonary disease	1 (5%)
Diabetes mellitus	1 (5%)
Hypertension	11 (55%)
Hypercholesterolemia	12 (60%)
Family history of coronary artery disease	5 (39%) ***
Prior myocardial infarction	3 (15%)
Prior percutaneous coronary intervention	3 (15%)
Prior bypass graft surgery	0 (0%)
Prior stroke	1 (5%)
Laboratory measures	
Total cholesterol, (mmol/L)	5.0 (4.2–6.4) ****
High-density lipoprotein, (mmol/L)	1.1 (1.0–1.3) *****
Low-density lipoprotein, (mmol/L)	2.8 (2.2–4.0) *****
Triglycerides, (mmol/L)	1.9 (1.2–2.6) *****
Hemoglobin, (mmol/L)	9.3 (8.7–10.1)
Leucocytes, (×10^9^/L)	7.7 (6.2–11.4) ******
Thrombocytes, (×10^9^/L)	204 (188–221)
Lipid-lowering medication prior to admission	9 (45%)
Statin	9 (45%)
Ezetimibe	0 (0%)
PCSK9 inhibitor	0 (0%)

Numbers are presented as median (interquartile range) or numbers (percentages). PCSK9 denotes proprotein convertase subtilisin/kexin type 9. * Data available from 11 patients. ** Data available from 15 patients. *** Data available from 13 patients. **** Data available from 7 patients. ***** Data available from 6 patients. ****** Data available from 19 patients.
